# Recent Patents of Interest to Dentists

**Published:** 1906-12

**Authors:** 


### RECENT PATENTS OF INTEREST TO DENTISTS.

833,883, to John A. Lentz, Phœnix, Ariz, Ter. Forming dental structures.

834,515, to Ernest D. R. Garden, Tarrytown N. Y. Artificial denture.

834,676, to Herman C. G. Luyties, St. Louis, Mo. Dentifrice.

834,594, to Crittenden Van Wyck, San Francisco, Cal. Obtunding apparatus.

834,790, to Wm. C. Wolford, Comfluence, Pa. Dental and Surgeons' cabinet.

834,899, to Wallace W. Freeman, Norfolk, Va. Dental handpiece.

835,431, to Henry A. Hughes, St. Louis, Mo. Projector for pathological work.

835,432, to Henry A. Hughes, St. Louis, Mo. Rubber-dam or bib holder.

835,358, to Thomas F. Kennedy, Janesville, Wis. Rubber-dam adjuster.

835,365, to Robert W. Morgan, Youngstown, Ohio. Combined toothbrush and holder.

838,305, to John H. Goss, Waterbury, Conn. Design, Top for powder receptacles.

836,001, to John E. Argue, Red Lake Falls, Minn. Dental tool.

835,732, to Wm. E. Lawrence, Minersville, Pa. Tooth-brush holder.

835,861, to Israel W. Lyon, Englewood, N. J. Tooth-powder cup.

835,709, to Chauncey D. Miller, Poughkeepsie, N. Y. Tooth-brush.

835,628, to Joseph Miller, Boxhill, England. Instrument for softening dental trial-plates.

Copies of these patents may be obtained for ten cents each, by addressing John A. Saul, Solicitor of Patents, Fendall Building, Washington, D. C.

